# Novel [FeFe]-Hydrogenase Mimics: Unexpected Course of the Reaction of Ferrocenyl α-Thienyl Thioketone with Fe_3_(CO)_12_ †

**DOI:** 10.3390/ma15082867

**Published:** 2022-04-14

**Authors:** Ahmad Q. Daraosheh, Hassan Abul-Futouh, Natsuki Murakami, Karl Michael Ziems, Helmar Görls, Stephan Kupfer, Stefanie Gräfe, Akihiko Ishii, Małgorzata Celeda, Grzegorz Mlostoń, Wolfgang Weigand

**Affiliations:** 1 Department of Chemistry, College of Arts and Sciences, University of Petra, P.O. Box 961343, Amman 11196, Jordan; adaraosheh@uop.edu.jo; 2 Department of Chemistry, Faculty of Science, The Hashemite University, P.O. Box 330127, Zarqa 13133, Jordan; 3 Department of Chemistry, Graduate School of Science and Engineering, Saitama University, Shimo-okubo, Sakura-ku, Saitama 338-8570, Japan; n.murakami.808@outlook.jp (N.M.); ishiiaki@chem.saitama-u.ac.jp (A.I.); 4 Institut für Physikalische Chemie und Abbe Center of Photonics, Friedrich-Schiller-Universität Jena, Helmholtzweg 4, 07743 Jena, Germany; karl-michael.ziems@uni-jena.de (K.M.Z.); stephan.kupfer@uni-jena.de (S.K.); s.graefe@uni-jena.de (S.G.); 5 Institut für Anorganische und Analytische Chemie, Friedrich-Schiller-Universität Jena, Humboldt Str. 8, 07743 Jena, Germany; helmar.goerls@uni-jena.de; 6 Department of Organic & Applied Chemistry, University of Lodz, Tamka 12, 91-403 Łódź, Poland; malgorzata.celeda@chemia.uni.lodz.pl

**Keywords:** sulfur–iron clusters, hydrogenase active centers mimics, heteroaryl thioketones, ferrocenyl thioketones, iron carbonyls, dearomatization, reaction mechanisms, cyclic voltammetry, X-ray diffraction analysis

## Abstract

The influence of the substitution pattern in ferrocenyl α-thienyl thioketone used as a proligand in complexation reactions with Fe_3_(CO)_12_ was investigated. As a result, two new sulfur–iron complexes, considered [FeFe]-hydrogenase mimics, were obtained and characterized by spectroscopic techniques (^1^H, ^13^C{^1^H} NMR, IR, MS), as well as by elemental analysis and X-ray single crystal diffraction methods. The electrochemical properties of both complexes were studied and compared using cyclic voltammetry in the absence and in presence of acetic acid as a proton source. The performed measurements demonstrated that both complexes can catalyze the reduction of protons to molecular hydrogen H_2_. Moreover, the obtained results showed that the presence of the ferrocene moiety at the backbone of the linker of both complexes improved the stability of the reduced species.

## 1. Introduction

**Figure 1:**
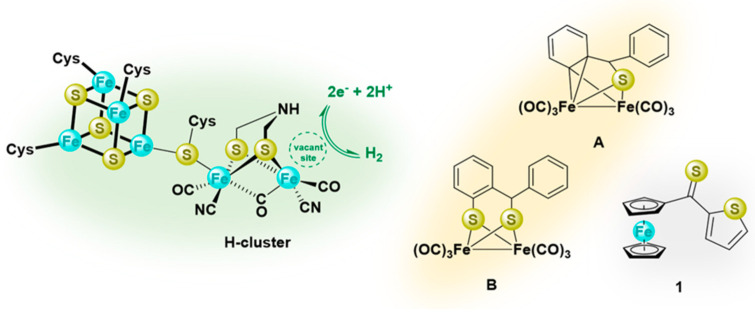
Presentation of the H-cluster (**left**); sulfur−iron complexes derived from thioketones, commonly considered models of the active site of [FeFe]−hydrogenase (middle, **A** and **B**), and structural formula of ferrocenyl α-thienyl thioketone (**1**) (**right**).

Multidisciplinary search for sustainable energy sources is considered a crucial task to the scientific community due to the environmental impact of non-renewable resources, such as fossil fuels, and limited access to gas and oil fields [1]. Therefore, hydrogen production without fossil fuels, so-called “Green hydrogen”, can play the role of a clean alternative as a promising fuel that substantially can reduce atmospheric pollution [2]. In this context, nature provides special metalloenzymes called hydrogenases that regulate the generation and depletion of hydrogen [3,4,5]. In particular, the [FeFe]-hydrogenases are the most competent hydrogen producer in nature with high turnover frequencies up to 10^4^ s^−1^ [6,7]. The active site of the latter, commonly referred to as the H-cluster, consists of a canonical [Fe_4_S_4_]-cluster coupled to an organometallic diiron complex, the [Fe_2_S_2_] subsite, which is coordinated with an azadithiolato bridging unit, by three carbon monoxide and two cyanide ligands (Figure 1) [8,9,10,11]. Based on that, the preparation of [FeFe]-hydrogenase mimics, which are used for electrocatalytic as well as photocatalytic production of hydrogen, has received increased attention in bioorganometallic chemistry [12,13,14,15,16,17,18,19,20,21,22,23]. Alterations of the bridge that connects the bimetallic atoms in those architectural mimics are considered a key factor in tuning its reduction potential and the reversibility of the catalytic reactions [12,24,25].

In recent publications, the reactions of thiobenzophenone (Ph_2_C=S) with triiron dodecacarbonyl Fe_3_(CO)_12_, leading to diverse iron sulfur clusters, were described, and in most cases, the [FeFe]-hydrogenase-mimicking complexes have been reported as major products [26,27]. The reaction performed with 1:1 ratio of thiobenzophenone and Fe_3_(CO)_12_ led to the *ortho*-metalated product **A** (Figure 1), which subsequently reacted with an equivalent of thioketone with the formation of complex **B** (Figure 1) [27]. Only in recent years were the relatively little known heteroaryl thioketones, bearing the α-thienyl substituent, widely explored as versatile substrates in the current organic synthesis designed for the preparation of diverse sulfur-containing compounds, and (3 + 2)- as well as (4 + 2)-cycloaddition reactions are the focus of interest [28]. For example, they smoothly undergo (3 + 2)-cycloadditions with nitrilimines and thiocarbonyl S-methanides with regioselective formation of 1,3,4-thiadiazoles and 1,3-dithiolanes, respectively [29,30,31]. The reactions of thioketones with diazomethane deserve special attention; they were shown to occur at low temperature with evolution of N_2_, and the in situ-generated thiocarbonyl *S*-methanides underwent a unique, step-wise dimerization leading to 14-membered sulfur macrocycles [32]. Furthermore, their formation was explained by the appearance of transient species presented as delocalized 1,7-diradicals. Later, this hypothesis was confirmed by a computational study [33]. An analogous, stepwise reaction course was postulated for reactions of α-thienyl substituted thioketones with other diazo compounds, such as trimethylsilyldiazomethane TMSCHN_2_ and 2-diazopropane Me_2_CN_2_ [34]. Notably, a stepwise mechanism was also postulated for the trienamine-mediated *hetero*-Diels-Alder reactions of some heteroaryl thioketones with 2,4-dienals [35]. Analogously, *hetero*-Diels-Alder reactions of di(α-thienyl) thioketone with non-activated dienes were shown to occur step-wise via a diradical intermediate [36].

Motivated by our results obtained with thiobenzophenone with Fe_3_(CO)_12_ as well as cycloaddition reactions of heteroaryl thioketones, we decided to investigate the use of ferrocenyl α-thienyl thioketone (**1**) (Figure 1) as a novel proligand for the synthesis of [FeFe]-hydrogenase H-cluster mimics. The expected sulfur–iron complexes should be characterized using a variety of spectroscopic techniques (NMR, MS, IR), elemental analysis, as well as by X-ray analysis. Moreover, the electrochemical properties of the obtained complexes were studied by cyclic voltammetry in combination with quantum chemical simulations.

## 2. Materials and Methods

### 2.1. General Methods

All reactions were performed under a neutral gas atmosphere (N_2_) using standard Schlenk and vacuum-line techniques. The ^1^H (400 MHz) and ^13^C{^1^H} (100 MHz) spectra were recorded with a Bruker Avance 400 MHz spectrometer (Terre Haute, IN, USA). Chemical shifts (ppm) refer to internal Me_4_Si (^1^H, ^13^C). The MS spectra were recorded using a Finnigan MAT SSQ 710 (Auburn, CA, USA) instrument. Elemental analyses were carried out using a Leco CHNS-932 (Madrid, Spain) analyzer. TLC tests were performed using Merck TLC aluminum sheets coated with silica (Silica gel 60 F254, Kenilworth, NJ, USA). Solvents and chemicals were purchased from commercial suppliers (Fisher Scientific, Acros Organics, Janssen-Pharmaceuticalaan, Belgium and Sigma-Aldrich, St. Louis, MO, USA) and they were used without further purification; solvents were dried and distilled prior to usage based on standard methods. Ferrocenyl α-thienyl thioketone (**1**) was synthesized following the known literature method [37]. Electrochemical measurements, computational work, and X-ray structure data for complexes **1** and **2** can be found in the Supplementary Materials (p. 3).

### 2.2. Synthesis of Complexes ***2*** and ***3***

A 100-mL oven-dried round-bottom Schlenk flask was charged with Fe_3_(CO)_12_ (133 mg, 0.265 mmol) and thioketone **1** (248 mg, 0.795 mmol), and then 30 mL dry THF was added. The obtained solution was heated under a reflux condenser for 4 h under an inert gas (nitrogen) atmosphere. After this time, the brown solution was filtered to remove a black solid material, the solvent was evaporated, and the residue was purified by column chromatography on silica using hexane/CH_2_Cl_2_ (2:1) as an eluent. Complex **2** was collected as a less polar fraction, while complex **3** was isolated as a more polar one.

Complex **2**. (27% yield, red-orange powder); Anal. Calcd for C_21_H_12_Fe_3_O_6_S_2_: C, 42.61; H, 2.04, S, 10.83. Found: C, 42.41; H, 2.24; S, 10.55. FT-IR (solid state, ATR, cm^−1^): *ν*_C__≡O_ 2069, 2026, 1969. ^1^H NMR (400 MHz, CD_2_Cl_2_, ppm): *δ* = 7.52 (d, 1H, ^2^*J*_H,H_ = 5.0 Hz), 7.49 (d, 1H, ^2^*J*_H,H_ = 5.0 Hz), 5.43 (s, 1H), 4.19 (s, 5H), 4.10 (s, 2H), 3.96 (s, 1H), 3.03 (s, 1H). ^13^C{^1^H} NMR (100 MHz, CD_2_Cl_2_, ppm): *δ* = 210.2, 210.1, 161.6, 142.4, 131.6, 120.6, 91.6, 69.9, 69.2, 67.9, 66.6, 58.0. DEI-MS (m/z): 591 [M]^+^, 563 [M−CO]^+^, 535 [M−2CO]^+^, 507 [M−3CO]^+^, 479 [M−4CO]^+^, 451 [M−5CO]^+^ and 423 [M−6CO]^+^.

Complex **3**. (30% yield, green powder); Anal. Calcd for C_36_H_24_Fe_4_O_6_S_3_: C, 49.58; H, 2.77, S, 11.03. Found: C, 49.43; H, 2.51; S, 10.98. FT-IR (solid state, ATR, cm^−1^): *ν*_C__≡O_ 2068, 2035, 1994. ^1^H NMR (400 MHz, CDCl_3_, ppm): *δ* = 7.47–7.14 (m, 3H), 6.88 (dd, 1H, ^3^*J*_H,H_ = 15.0/12.0 Hz), 6.68 (d, 1H, ^3^*J*_H,H_ = 12.0 Hz), 6.49 (d, 1H, ^3^*J*_H,H_ = 15.0 Hz), 4.46, 4.40, 4.34, 4.24, 4.19 (s, 18H). ^13^C{^1^H} NMR (100 MHz, CDCl_3_, ppm): *δ* = 208.8, 149.7, 145.0, 139.9, 137.4, 131.9, 128.9, 128.0, 127.3, 127.1, 126.5, 87.2, 81.7, 70.9, 70.4, 70.3, 69.5, 67.9, 67.5. DEI-MS (m/z): 872 [M]^+^, 844 [M−CO]^+^, 816 [M−2CO]^+^, 788 [M−3CO]^+^, 760 [M−4CO]^+^, 732 [M−5CO]^+^ and 704 [M−6CO]^+^.

## 3. Results and Discussion

### 3.1. Synthesis and Characterization of the Binuclear Complexes

**Scheme 1:**
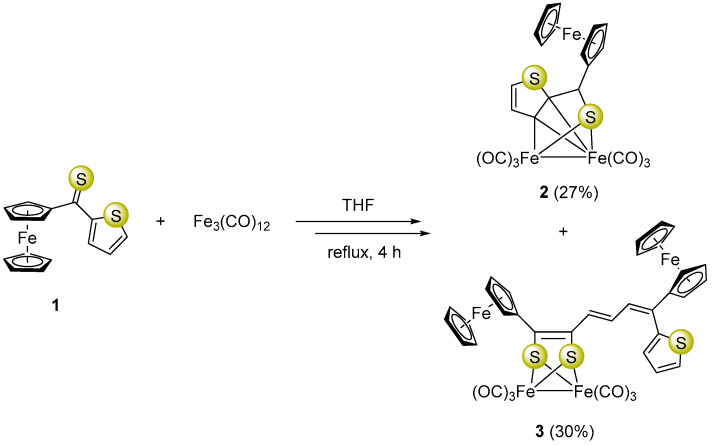
Formation of a mixture of complexes **2** and **3** starting with ferrocenyl α-thienyl thioketone (**1**) and Fe_3_(CO)_12_.

Synthesis of ferrocenyl α-thienyl thioketone (**1**), used in this study, was carried out following a previously reported procedure [37]. Heating of Fe_3_(CO)_12_ with three mol-equivalents of **1** in boiling THF for 4 h led to a mixture of comparable amounts of the *ortho*-metalated complex **2** and unexpectedly formed complex **3** in which a unique dearomatization of the thiophene ring was observed (Scheme 1).

**Scheme 2:**
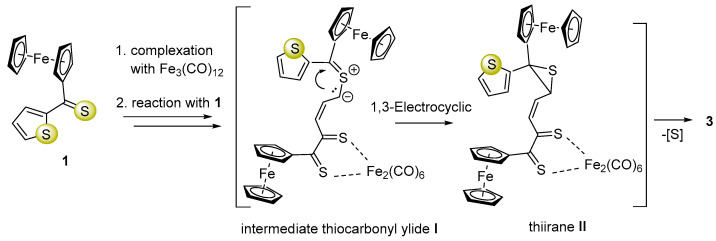
Plausible mechanism of multi-step reaction leading to complex **3** via thiocarbonyl ylide (**I**) and thiirane (**II**) postulated as a key intermediate.

The *ortho*-metallated complex **2** could be formed via a mechanism proposed earlier by Alper and co-workers [38]. However, the mechanism of the multi-step reaction leading to complex **3** is more complicated compared to that of **2** and comprises dearomatization of a five-membered heterocycle. We assume that this conversion is initiated by a ring-opening in **1** followed by an attack of the thiocarbonyl group from another equivalent of **1** forming a new S-C bond, which leads to in situ formation of thiocarbonyl ylide **I** (1,3-dipole) as a reactive intermediate [39] (Scheme 2). The latter subsequently undergoes electrocyclic ring closure with exclusive formation of thiirane (**II**). This reaction sequence terminates with elimination of sulfur and offers a plausible explanation of the mechanisms of this unexpected reaction leading to dearomatization of the thiophene ring and formation of complex **3** as the final product (Scheme 2).

The structures of **2** and **3** were established by spectroscopic methods (^1^H NMR, ^13^C{^1^H} NMR, IR, MS) and elemental analysis and were unambiguously confirmed by X-ray single crystal diffraction analysis. For example, in the IR spectra of both complexes, three characteristic metal/carbonyl stretching bands located at 2069, 2026, 1969 for **2**, and at 2068, 2035, 1994 cm^−1^ for **3** were observed. These results are consistent with the IR data reported for similar iron complexes [26,27,38,39,40]. The ^1^H NMR spectrum of **2** displays a singlet resonance at 5.43 ppm for the methine proton. Moreover, the nine protons of the ferrocene moiety appear as four singlets in the area between 3.03 and 4.19 ppm, while those of the thiophene ring were found as two doublets (AB spin system) at *δ* 7.52 and 7.49 ppm (^2^*J*_H,H_ = 5.0 Hz). In the case of complex **3**, five singlet signals were detected in the range of *δ* 4.19–4.46 ppm, which can be assigned to the 18 protons of two ferrocene moieties. The three protons of the diene moiety were detected as two doublets at 6.68 (^3^*J*_H,H_ = 12.0 Hz) and 6.49 ppm (^3^*J*_H,H_ = 15.0 Hz) and as a doublet of the doublet at 6.88 ppm (^3^*J*_H,H_ = 12.0/15.0 Hz). In the ^13^C{^1^H} NMR spectra, the terminal carbonyl *C*-atoms of the iron cores of complexes **2** and **3** resonated as a singlet at *δ* 210.2 and 210.1 and at *δ* 208.8 ppm, respectively. The signals of the other carbon atoms, which confirmed the postulated structures of complexes **2** and **3**, were also detected in the expected range.

### 3.2. Molecular Structures of Complexes ***2*** and ***3***

**Figure 2:**
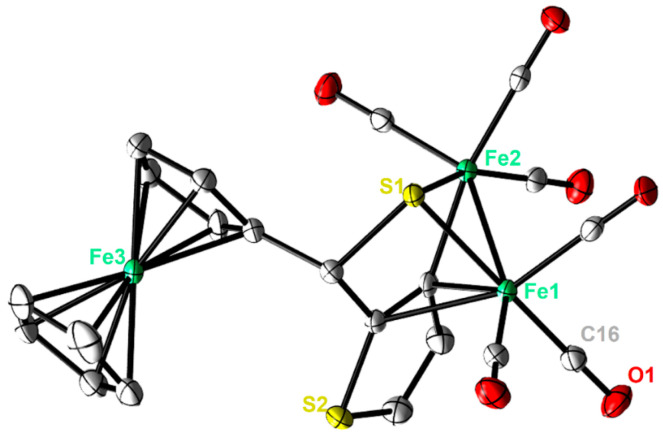
Molecular structures (50% probability) of **2**. All hydrogen atoms are excluded for clarity.

**Figure 3:**
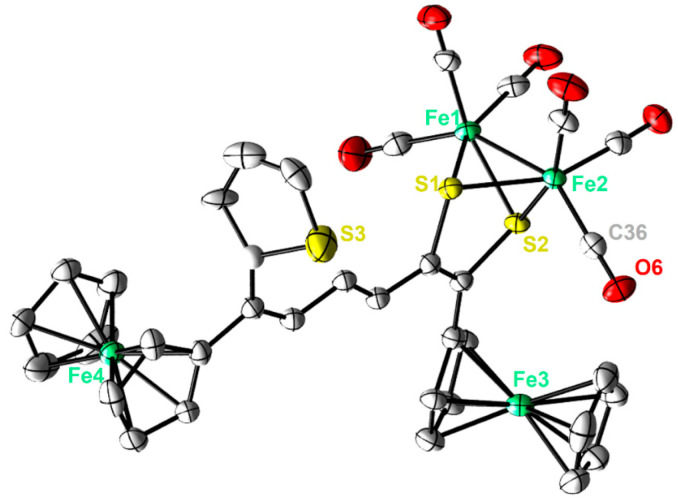
Molecular structures (50% probability) of **3**. All hydrogen atoms are excluded for clarity.

Single crystals of complexes **2** and **3** adequate for X-ray diffraction measurement were grown at low temperature (−20 °C) via diffusion of pentane into a dichloromethane solution of each complex. Figure 2 and Figure 3 illustrate the molecular shapes of complexes **2** and **3**, respectively. The thiophene ring in **3** is disordered over two positions. It is evident from Figure 2 that the ferrocenyl α-thienyl thioketone ligand is connected to the two iron atoms via the sulfur atom, with an average Fe-S bond length of 2.2610 Å. Moreover, it is also σ bounded to one iron atom through the β-carbon of the thiophene ring and is π-bounded to the other iron atom through one C-C π-bond. The Fe–Fe (2.5036(6) Å) bond length of complex **2** is in good agreement with those for similar *ortho*-metalated analogues [26,27]. The two symmetric cyclopentadienyl rings exhibit an eclipsed configuration. On the other hand, the solid-state structure of **3** reveals the usual butterfly conformation typical for the synthetic H-cluster mimics, indicating a distorted octahedron geometry for each iron atom [41,42,43,44]. The Fe–Fe bond length (2.4793(8) Å) of **3** is slightly shorter than those described for analogues models [12,13,14,15,16,17,41,42,43,44]. The interatomic S···S distance (2.9080(1) Å) in complex **3** is slightly shorter than the theoretical value calculated for similar binuclear dithiolato complexes [45] and in good agreement with the present simulations (2.9191 Å). In comparison to complex **2**, the ferrocene moieties of complex **3** show also an eclipsed conformation. The torsion angle defined by the apical CO ligand across the Fe–Fe vector in complex **3** is almost zero, indicating that the Fe(CO)_3_ units eclipsed each other. The average Fe–S bond length (2.2668 Å) is within the same range as those observed for its analogues. The average Fe–CO bond length in **3** is 1.8033 Å, which agrees with data reported for similar [FeFe]-hydrogenase mimics [46,47,48].

### 3.3. Electrochemical Investigation

**Figure 4:**
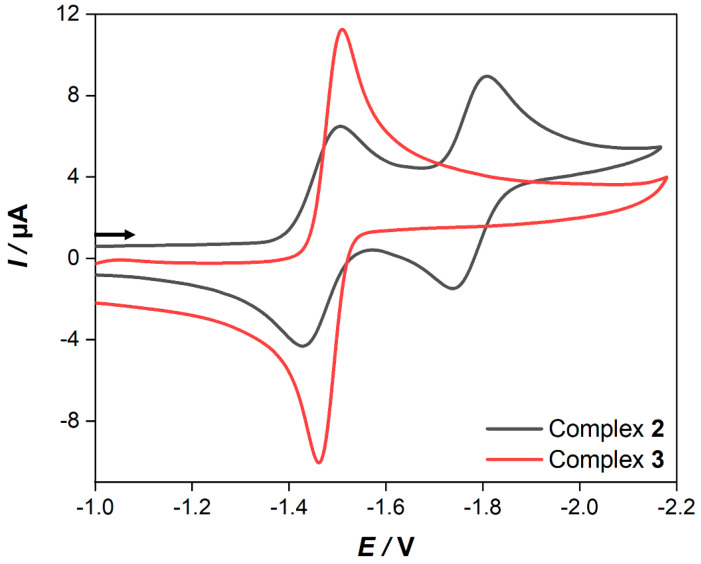
Cyclic voltammetry of 1.0 mM solutions of complexes **2** and **3** in CH_2_Cl_2_−[*n*−Bu_4_N][BF_4_] (0.1 M) registered at a 0.2 V/s scan rate. The arrow indicates the scan direction. The potentials *E* are given in V and are referenced to the Fc^+^/Fc couple.

In order to gain more insight into the redox properties of **2** and **3**, cyclic voltammetry (CV) experiments were performed in 0.1 M CH_2_Cl_2_-[*n*-Bu_4_N][BF_4_] solutions. The obtained CVs of **2** and **3** at a scan rate of 0.2 V/s are exhibited in Figure 4.

**Figure 5:**
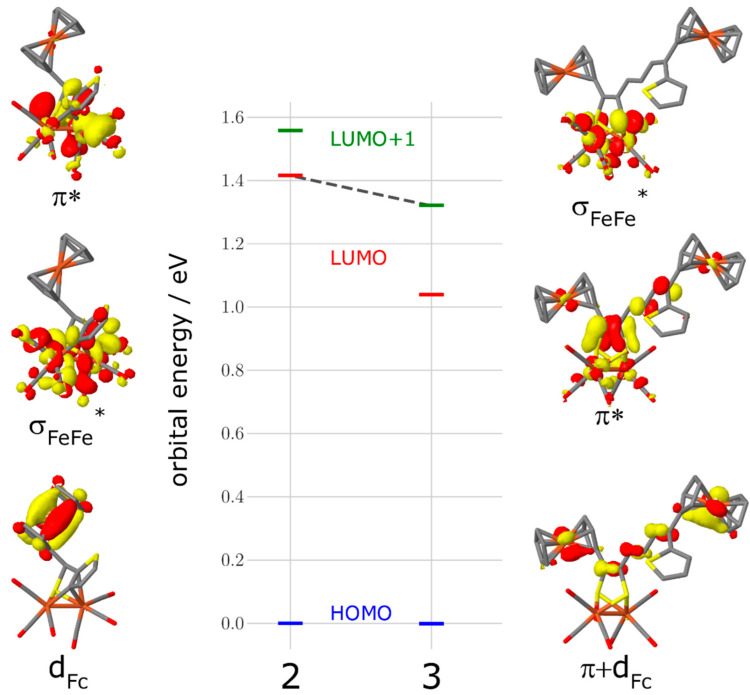
Orbital energy and orbital pictures of the HOMO, LUMO, and LUMO+1 for complex **2** (left) and complex **3** (right). The grey dashed line between complex **2**-LUMO and complex **3**-LUMO+1 highlights the change in the orbital order for the relevant σ* character orbital at the active [Fe–Fe] center.

As apparent from Figure 4, the CV of **2** reveals two consecutive, reversible, one-electron steps at *E*_1/2_ = −1.47 V and *E*_1/2_ = −1.78 V, respectively, while in the case of **3**, there is only one reversible reduction peak found at *E*_1/2_ = −1.49 V. A plot of the current function (*I*_pc_/*C*·*ν*^1/2^) (*I*_pc_ = cathodic peak current, *C* = concentration and *ν* = scan rate) vs. the scan rates of the reduction event of **3** reveals the expected invariance for a process involving the transfer of two electrons (Figure S1) [49,50,51,52,53]. Additionally, the value of *I*_pc_*/C·ν*^1/2^ for the reduction peak of **3** is almost twice the magnitude of that for the first reduction event of **2**. According to this, the reduction event of complex **3** is due to the transfer of two electrons in a single step via an ECE mechanism (E = electrochemical process, C = chemical process). Furthermore, when the scan rate is increased to 10 V/s, these reduction events appear chemically reversible (Figure S2). In addition, their currents vs. the square root of the scan rates are all linearly correlated, which indicates that these reduction processes are diffusion-controlled (Figure S3) [54]. It is worth pointing out that the backbone of the dithiolate linker of complex **3** enhances the chemical stability of the reduced species, which prevents the formation of any dimerization process such as the case in the [Fe_2_(CO)_6_{μ-pdt}] (pdt = 1,3-propane dithiolate) complex [41]. However, this might be explained due to the flexibility of the ferrocene moiety as well as the delocalization of the negative charge onto the backbone of the dithiolato linker similar to the behavior of the reported [Fe_2_(CO)_6_{μ-bdt}] (bdt = 1,2-benzenedithiole) complex [55,56,57]. To further understand the reduction processes in complex **2** and **3**, we show the complexes’ frontier orbitals in Figure 5, namely, the highest occupied molecular orbital (HOMO), the lowest unoccupied orbital (LUMO), and the LUMO+1 as obtained at the density functional level of theory (DFT). For complex **2**, the HOMO is of d-orbital character at the ferrocene unit, while the LUMO is localized at the [Fe–Fe] center (σ* character) with a gap of 1.42 eV. In contrast, the HOMO and LUMO of complex **3** with a gap of 1.04 eV are of π/π* character on the conjugated ligand. Contrary to complex **2**, the orbital of σ* character at the [Fe–Fe] center is now the LUMO+1 (see grey dashed line, Figure 5). Hence, we expect the reduction of complex **2** via the LUMO to lead to substantial electron density at the active [Fe–Fe] center–formally yielding a [Fe^I^-Fe^0^] species—while for complex **3**, the reduction via the LUMO will transfer electron density on the ligand site (π* character). This opens up complex **2** as an interesting candidate to mimic the reduction process seen at the [Fe–Fe] center in hydrogenases.

On initiating the electrochemical scan in the positive direction at a scan rate of 0.2 V/s, complex **2** exhibits the appearance of a quasi-reversible oxidation peak at *E*_1/2_ = 0.09 V. In the case of complex **3**, it shows two sequential quasi-reversible one electron steps at *E*_1/2_ = 0.03 and 0.13 V. These oxidation events could be assigned to the one-electron oxidation process of Fe(II) → Fe(III) of the ferrocene moiety.

### 3.4. Electrocatalysis

**Figure 6:**
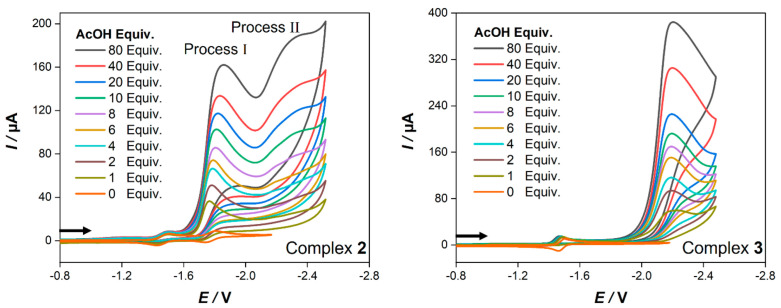
Cyclic voltammetry (0.2 V/s) of 1.0 mM of complexes **2** (left) and **3** (right) in CH_2_Cl_2_−[*n*−Bu_4_N][BF_4_] (0.1 M) in the presence of different equivalents of AcOH. Potential *E* is given in volts V and is referenced to the Fc^+^/Fc couple. The arrows indicate the scan direction.

To estimate the ability of complexes **2** and **3** to catalyze the electrocatalytic hydrogen production, we measured their cyclic voltammograms in the presence of acetic acid as a source of protons. The CVs of both complexes recorded after the addition of 1–80 equiv. of a weak acid (acetic acid, AcOH) are illustrated in Figure 6. Under the same experimental conditions, AcOH is reduced at a potential of −2.58 V in the absence of complexes **2** and **3** (Figure S4).

It is clear from Figure 6 that the voltammogram of **2** exhibits catalytic activity through two processes at −1.77 V (process I) and at −2.18 V (process II) while complex **3** shows only one catalytic process at −2.22 V. After the addition of one equiv. AcOH to a solution of complex **2**, the current and the potential of the first reduction peak (*E*_pc_ = −1.50 V) are hardly affected, and it remains reversible at all acid concentration (Figure S5). Accordingly, the potential of the second reduction event (*E*_pc_ = −1.81 V) shows a small anodic shift (40 mV), and its current increased dramatically with sequential adding of AcOH (Figure 6). This suggests that at this potential, an electrocatalytic proton reduction to hydrogen is catalyzed by complex **2**. Furthermore, a new reduction peak starts to appear in the presence of 6 equiv. AcOH near ~−2.18 V, and its current increases in response to the systematic increase of AcOH. Based on these findings, we might postulate the mechanism of electrocatalytic hydrogen evolution by complex **2** (process I) as follows: complex **2** is first reduced at −1.50 V to generate the monoanionic species {**2^−^**}, which then accepts another electron at −1.77 V to yield the dianionic species {**2^2−^**}. Afterwards, the latter undergoes protonation by AcOH to yield the {**2H^−^**} species, that subsequently accepts another proton to evolve H_2_ and regenerates complex **2** to close the catalytic cycle. The mechanism of process II may begin with the reduction of {**2H^−^**} to generate the {**2H^2−^**} species. Further protonation leads to H_2_ evolution and restores {**2^−^**} species to end the catalytic cycle. However, the electrocatalytic reduction mechanism of acetic acid by complex **2** is comparable to that in the case of its analogue [Fe_2_(CO)_6_{к,μ-S,η^2^-(C_13_H_8_OS)}], and its catalytic events are shifted to less negative potentials (10 mV for process I and 20 mV for process II) [57]. On the other hand, the addition of one equiv. of AcOH to a solution of **3** results in an anodic shift (20 mV) of its single step two-electron reduction event as well as the disappearance of its reoxidation peak (*E*_pa_ = −1.46 V) (Figure S6). This suggests a protonation of the dianionic species {**3^2−^**} to generate {**3H^−^**} as was reported in the case of [Fe_2_(CO)_6_{μ-bdt}] [58]. This species then accepts another electron at −2.22 V (Figure 6) to yield the {**3H^2−^**} species, followed by protonation to yield H_2_ and releasing {**3^−^**} species to complete the catalytic cycle. This postulated electrocatalytic hydrogen production by complex **3** is consistent with those of its analogous models [58,59].

## 4. Conclusions

The study showed that the complexation of ferrocenyl α-thienyl thioketone with Fe_3_(CO)_12_ leads to a mixture of comparable amounts of two complexes that can be considered new mimics of the hydrogenase H-cluster. Whereas cluster **2** resembles similar structures of complexes obtained in analogous reactions of aromatic thioketones, complex **3** presents a completely new arrangement of sulfur and iron atoms, resulting from the ring opening, i.e., dearomatization of the thiophene ring, and this an unprecedented result is not published in the literature to date. In this unexpected process, a transient thiocarbonyl ylide is postulated as a key intermediate.

The analysis of cyclic voltammograms of both complexes indicates that **2** reacts via two successive one-electron processes (EE mechanism) and in the case of **3**, transfer of two electrons in a single step via the ECE mechanism was observed. The proton reduction processes in AcOH solution, catalyzed by **2** and **3**, were also studied by cyclic voltammetry and compared in terms of their efficiency. The presented results open a new perspective for further studies in this area focused on the potential of photo-induced electron transfer between the ligand and the [Fe–Fe] centered orbitals. In addition, the substitution of one CO ligand in complexes **2** and **3** by stronger, electron-donating ligands should enable a tuning of their redox properties. Potentially present Fe/S-clusters can be considered promising photosensitizers for catalytic hydrogen evolution reaction. In general, the study demonstrated the usefulness of ferrocenyl and hetaryl thioketones in current organic synthesis and especially their usefulness as starting materials for reactions with iron carbonyls aimed at the preparation of diverse sulfur–iron clusters that mimic [FeFe]-hydrogenase active centers.

## Data Availability

The data that support the findings of this study are available from the corresponding author upon reasonable request.

## Supplementary Materials

The following supporting information can be downloaded at: https://www.mdpi.com/article/10.3390/ma15082867/s1, Crystallographic data (excluding structure factors) have been deposited in the Cambridge Crystallographic Data Centre as supplementary publication CCDC-2110902 for **2** and CCDC-2110903 for **3**. Copies of the data can be obtained free of charge on application to CCDC, 12 Union Road, Cambridge CB2 1EZ, UK [E-mail: deposit@ccdc.cam.ac.uk]. Figure S1: Scan rate dependence of the current function of the reduction events of complexes **2** (black) and **3** (red); Figure S2: Cyclic voltammetry of 1.0 mM of complexes **2** and **3** in CH_2_Cl_2_-[*n*-Bu_4_N][BF_4_] (0.1 M) solution at various scan rates. The arrows indicate the scan direction. The potentials *E* are given in V and referenced to the Fc^+^/Fc couple; Figure S3: Plots of *I*_p_ versus *ν*^1/2^ for the first (•) and second (•) reduction peaks of **2** as well as reduction peak of **3** (•); Figure S4: Cyclic voltammogram of various concentration of AcOH in CH_2_Cl_2_-[*n*-Bu_4_N][BF_4_] (0.1 M) solution at 0.2 V/s scan rate in the absence of catalyst (complexes **2** and **3**). The arrow indicates the scan direction. The potentials *E* are given in V and referenced to the Fc^+^/Fc couple; Figure S5: Cyclic voltammetry (0.2 V/s) of 1.0 mM of complex **2** in CH_2_Cl_2_- [*n*-Bu_4_N][BF_4_] (0.1 M) in the presence of one equiv. AcOH. Potential *E* is given in volts V and referenced to Fc^+^/Fc couple. The arrow indicates the scan direction; Figure S6: Cyclic voltammetry (0.2 V/s) of 1.0 mM of complex **3** in CH_2_Cl_2_- [*n*-Bu_4_N][BF_4_] (0.1 M) in the presence of one equiv. AcOH. Potential *E* is given in volts V and referenced to Fc^+^/Fc couple. The arrow indicates the scan direction; Figure S7: ^1^H NMR spectrum of compound **2** in dichloromethane-d^2^ (400 MHz); Figure S8: ^13^C{^1^H} NMR spectrum of compound **2** in dichloromethane-d^2^ (400 MHz); Figure S9: ^1^H NMR spectrum of compound **3** in dichloromethane-d^2^ (400 MHz); Figure S10: ^13^C{^1^H} NMR spectrum of compound **3** in dichloromethane-d^2^ (400 MHz); Figure S11: Packing diagrams of complexes **2** (top) and **3** (bottom). References [60,61,62,63,64,65,66,67,68,69,70,71] are cited in the supplementary materials.
