# Supplementary material for: Novel [FeFe]-Hydrogenase Mimics: Unexpected Course of the Reaction of Ferrocenyl α-Thienyl Thioketone with Fe3(CO)12
Source: Materials (Basel). 2022 Apr 14;15(8):2867. doi: 10.3390/ma15082867 (PMC9029206; doi:10.3390/ma15082867)
Supplement: Supplementary file 1 [file materials-15-02867-s001.zip › materials-1542732-supplementary.pdf]

materials

MDPI

Article

Novel [FeFe]-Hydrogenase Mimics: Unexpected Course of the Reaction of Ferrocenyl α-Thienyl Thioketone with $Fe_3(CO)_{12}$

Ahmad Q. Daraosheh [1], Hassan Abul-Futouh [2,*], Natsuki Murakami [3], Karl Michael Ziems [4], Helmar Görls [5], Stephan Kupfer [4], Stefanie Gräfe [4], Akihiko Ishii [3], Małgorzata Celeda [6], Grzegorz Mlostoń [6,*] and Wolfgang Weigand [5,*]

[1] Department of Chemistry, College of Arts and Sciences, University of Petra, P.O. Box 961343, Amman 11196, Jordan; adaraosheh@uop.edu.jo
[2] Department of Chemistry, Faculty of Science, The Hashemite University, P.O. Box 330127, Zarqa 13133, Jordan
[3] Department of Chemistry, Graduate School of Science and Engineering, Saitama University, Shimo-okubo, Sakura-ku, Saitama 338-8570, Japan; n.murakami.808@outlook.jp (N.M.); ishiiaki@chem.saitama-u.ac.jp (A.I.)
[4] Institut für Physikalische Chemie und Abbe Center of Photonics, Friedrich-Schiller-Universität Jena, Helmholtzweg 4, 07743 Jena, Germany; karl-michael.ziems@uni-jena.de (K.M.Z.); stephan.kupfer@uni-jena.de (S.K.); s.graefe@uni-jena.de (S.G.)
[5] Institut für Anorganische und Analytische Chemie, Friedrich-Schiller-Universität Jena, Humboldt Str. 8, 07743 Jena, Germany; helmar.goerls@uni-jena.de
[6] Department of Organic & Applied Chemistry, University of Lodz, Tamka 12, 91-403 Łódź, Poland; malgorzata.celeda@chemia.uni.lodz.pl
* Correspondence: habulfutouh@gmail.com (H.A.-F.); grzegorz.mloston@chemia.uni.lodz.pl (G.M.); wolfgang.weigand@uni-jena.de (W.W.)

Citation: Daraosheh, A.Q.; Abul-Futouh, H.; Murakami, N.; Ziems, K.M.; Görls, H.; Kupfer, S.; Gräfe, S.; Ishii, A.; Celeda, M.; Mloston, G.; et al. A Novel Type of the [FeFe]-Hydrogenase Mimics: Unexpected Course of the Reaction of Ferrocenyl α-Thienyl Thioketone with $Fe_3(CO)_{12}$. *Materials* **2022**, *15*, 2862. https://doi.org/10.3390/ma15082862

Academic Editor: Maria Rosaria Plutino

Received: 24 February 2022
Accepted: 11 April 2022
Published: 13 April 2022

Publisher's Note: MDPI stays neutral with regard to jurisdictional claims in published maps and institutional affiliations.

Copyright: © 2022 by the authors. Submitted for possible open access publication under the terms and conditions of the Creative Commons Attribution (CC BY) license (https://creativecommons.org/licenses/by/4.0/).

Abstract: The influence of the substitution pattern in ferrocenyl α-thienyl thioketone used as a proligand in complexation reactions with $Fe_3(CO)_{12}$ was investigated. As a result, two new sulfur–iron complexes, considered [FeFe]-hydrogenase mimics, were obtained and characterized by spectroscopic techniques (^1H, $^{13}C\{^1H\}$ NMR, IR, MS), as well as by elemental analysis and X-ray single crystal diffraction methods. The electrochemical properties of both complexes were studied and compared using cyclic voltammetry in the absence and in presence of acetic acid as a proton source. The performed measurements demonstrated that both complexes can catalyze the reduction of protons to elemental hydrogen H_2. Moreover, the obtained results showed that the presence of the ferrocene moiety at the backbone of the linker of both complexes improved the stability of the reduced species.

Keywords: sulfur–iron clusters; hydrogenase active centers mimics; heteroaryl thioketones; ferrocenyl thioketones; iron carbonyls; dearomatization; reaction mechanisms; cyclic voltammetry; X-ray diffraction analysis

1. Electrochemistry

Corrections for the *iR* drop were performed for all experiments. Cyclic voltammetry measurements were conducted by applying the three-electrode technique - glassy carbon disk (diameter = 1.6 mm) as working electrode; Ag/Ag^+ in MeCN as reference electrode; Pt wire as counter electrode. The Reference 600 Potentiostat (Gamry Instruments) was used to realize the measurements. All experiments were performed at rt in CH_2Cl_2 solutions (concentration 1.0 mM), containing 0.1 M tetrabutylammonium tetrafluoroborate (n-Bu_4N)(BF_4). The solutions were initially purged with a gentle N_2 stream which subsequently was maintained over the solution surface during the measurement. Typically, the vitreous carbon disk was polished on a felt tissue coated with

alumina before the measurement started. All potential values reported in this paper are referenced to the potential of the ferrocenium/ferrocene (Fc^+/Fc) couple.

2. Computational Work

Quantum chemical calculations were carried out using the Gaussian 16 program [1], which is based on the DFT method by means of the hybrid functional B3LYP [2,3] and the triple-ς-valence-polarization (TZVP) [4] basis set. For the iron atoms, the effective core potential MDF10 [5] was used. Fully relaxed equilibrium structures of complexes 2 and 3 (singlet ground state) were obtained based on results of the X-ray analysis. A vibrational analysis upon geometry optimization confirmed the obtained structures by the presence of minima observed on the 3*N*-6-dimensional potential energy hypersurface.

3. X-Ray Crystal Structure Analysis

The intensity data for the compounds were collected on a Nonius KappaCCD diffractometer using graphite-monochromated Mo-K_α radiation. Data were corrected for Lorentz and polarization effects; absorption was considered on a semi-empirical basis using multiple-scans [6–8]. The structures were solved by direct methods (SHELXS) [9] and refined by full-matrix least squares techniques against Fo^2 (SHELXL-2018) [10]. All hydrogen atoms were included at calculated positions with fixed thermal parameters. Disordered thiophene molecule of 3 was refined using bond lengths restraints and displacement parameter restraints. The disorder model was introduced by the program DSR [11]. All non-hydrogen atoms were refined anisotropically [9] MERCURY was used for structure representations [12].

***Crystal Data for* 2:** $C_{21}H_{12}Fe_3O_6S_2$, Mr = 591.98 $gmol^{-1}$, brown prism, size 0.108 x 0.088 x 0.076 mm^3, monoclinic, space group C 2/c, a = 20.0130(5), b = 13.0408(3), c = 17.7418(4) Å, β = 111.899(1)°, V = 4296.24(18) $Å^3$, T = −140 °C, Z = 8, $\rho_{calcd.}$ = 1.830 gcm^{-3}, μ (Mo-K_α) = 22.3 cm^{-1}, multi-scan, transmin: 0.6461, transmax: 0.7456, F(000) = 2368, 14502 reflections in h(−25/25), k(−16/16), l(−23/22), measured in the range 2.194° ≤ Θ ≤ 27.481°, completeness Θmax = 99.4%, 4882 independent reflections, R_{int} = 0.0421, 4279 reflections with $F_o > 4\sigma(F_o)$, 289 parameters, 0 restraints, $R1_{obs}$ = 0.0328, wR^2_{obs} = 0.0612, $R1_{all}$ = 0.0408, wR^2_{all} = 0.0649, GOOF = 1.070, largest difference peak and hole: 0.440 / −0.345 e $Å^{-3}$.

***Crystal Data for* 3:** $C_{36}H_{24}Fe_4O_6S_3$, Mr = 872.13 $gmol^{-1}$, brown prism, size 0.102 x 0.100 x 0.066 mm^3, monoclinic, space group P 2_1/c, a = 7.6700(4), b = 38.7090(9), c = 11.4230(4) Å, β = 92.709(1)°, V = 3387.7(2) $Å^3$, T= −140 °C, Z = 4, $\rho_{calcd.}$ = 1.710 gcm^{-3}, μ (Mo-K_α) = 19.1 cm^{-1}, multi-scan, transmin: 0.5432, transmax: 0.7457, F(000) = 1760, 42623 reflections in h(−9/8), k(−50/50), l(−14/14), measured in the range 1.861° ≤ Θ ≤ 27.484°, completeness Θmax = 99.8%, 7745 independent reflections, R_{int} = 0.0485, 6047 reflections with $F_o > 4\sigma(F_o)$, 488 parameters, 76 restraints, $R1_{obs}$ = 0.0477, wR^2_{obs} = 0.1117, $R1_{all}$ = 0.0668, wR^2_{all} = 0.1196, GOOF = 1.029, largest difference peak and hole: 1.594 / −0.748 e $Å^{-3}$.

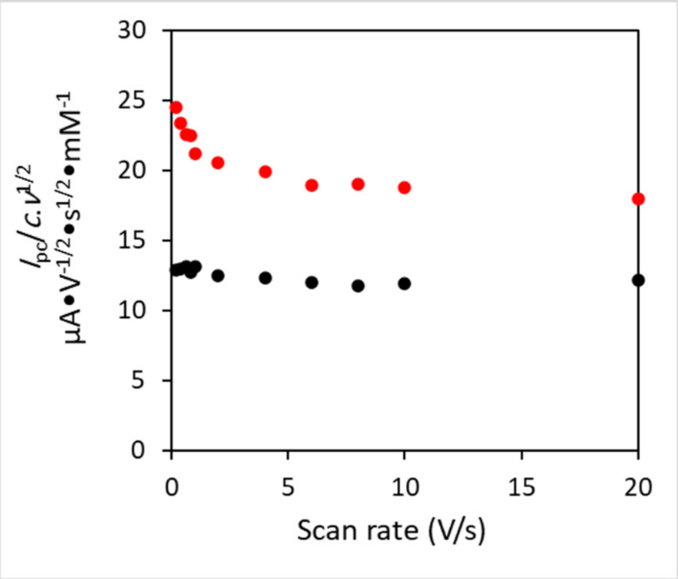

Figure S1. Scan rate dependence of the current function of the reduction events of complexes **2** (black) and **3** (red).

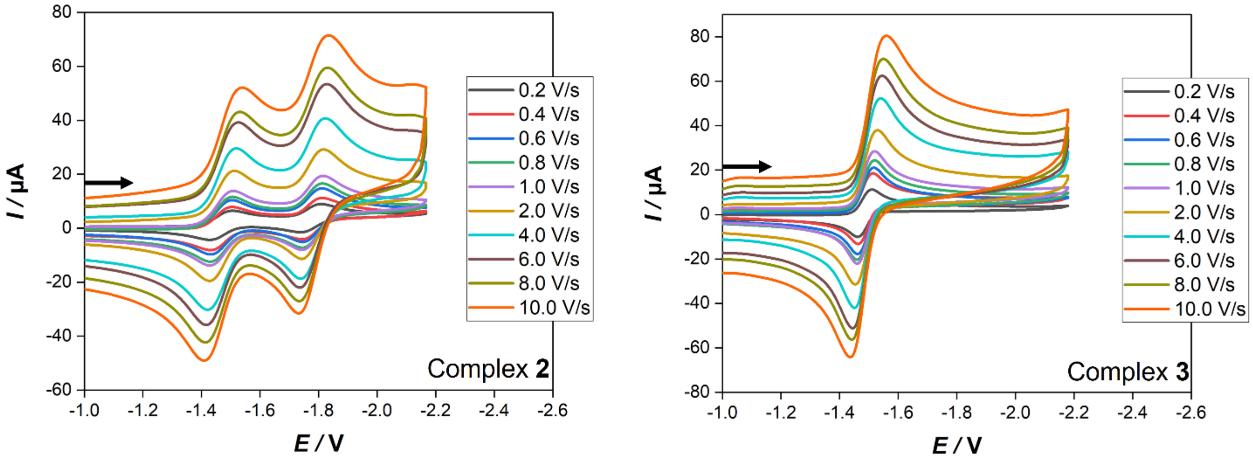

Figure S2. Cyclic voltammetry of 1.0 mM of complexes **2** and **3** in CH_2Cl_2–[*n*-Bu_4N][BF_4] (0.1 M) solution at various scan rates. The arrows indicate the scan direction. The potentials *E* are given in V and referenced to the Fc^+/Fc couple.

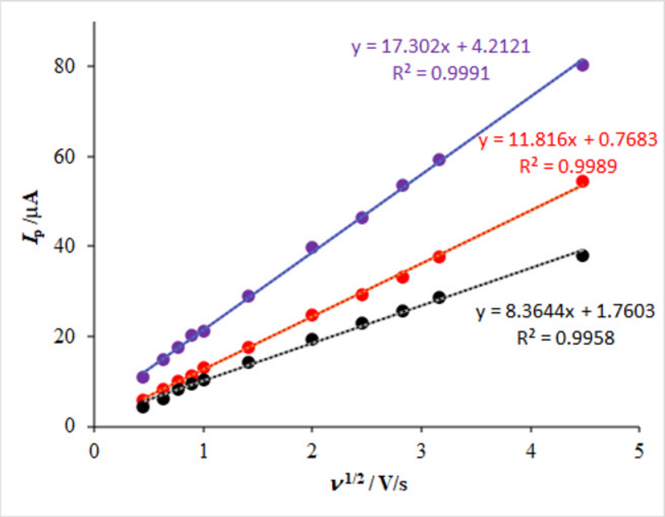

Figure S3. Plots of I_p versus $v^{1/2}$ for the first (•) and second (•) reduction peaks of **2** as well as reduction peak of **3** (•).

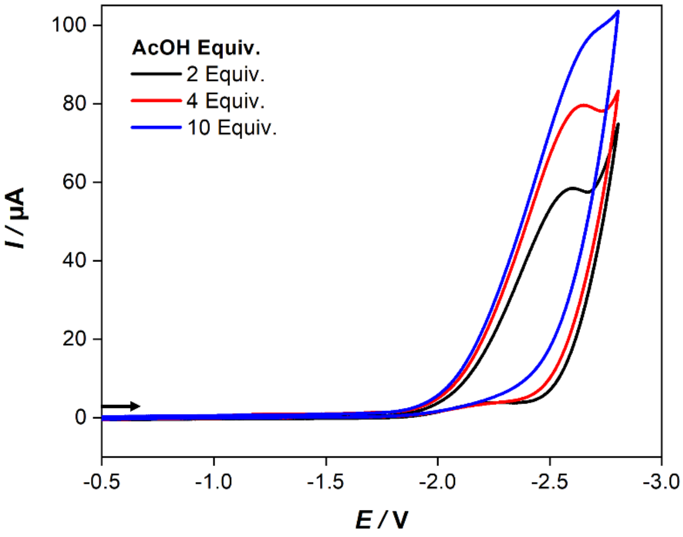

Figure S4. Cyclic voltammogram of various concentration of AcOH in CH_2Cl_2–[*n*-Bu_4N][BF_4] (0.1 M) solution at 0.2 V/s scan rate in the absence of catalyst (complexes **2** and **3**). The arrow indicates the scan direction. The potentials *E* are given in V and referenced to the Fc^+/Fc couple.

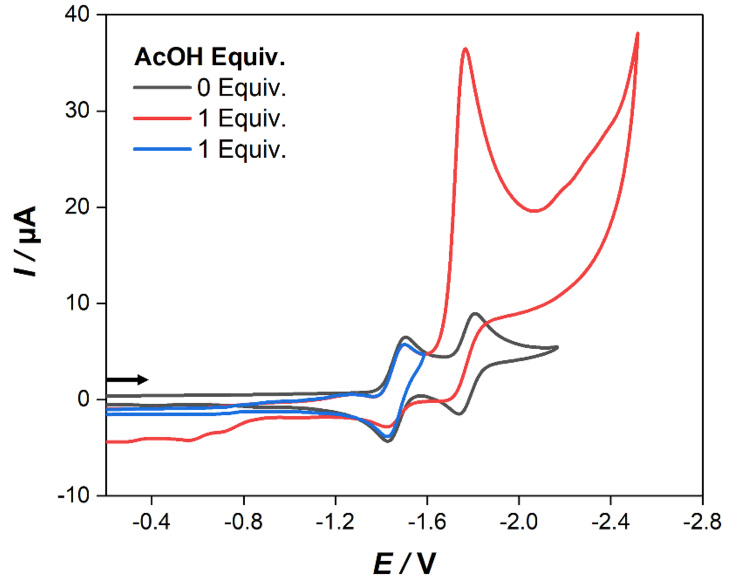

Figure S5. Cyclic voltammetry (0.2 V/s) of 1.0 mM of complex **2** in CH_2Cl_2– [*n*-Bu_4N][BF_4] (0.1 M) in the presence of one equiv. AcOH. Potential *E* is given in volts V and referenced to Fc^+/Fc couple. The arrow indicates the scan direction.

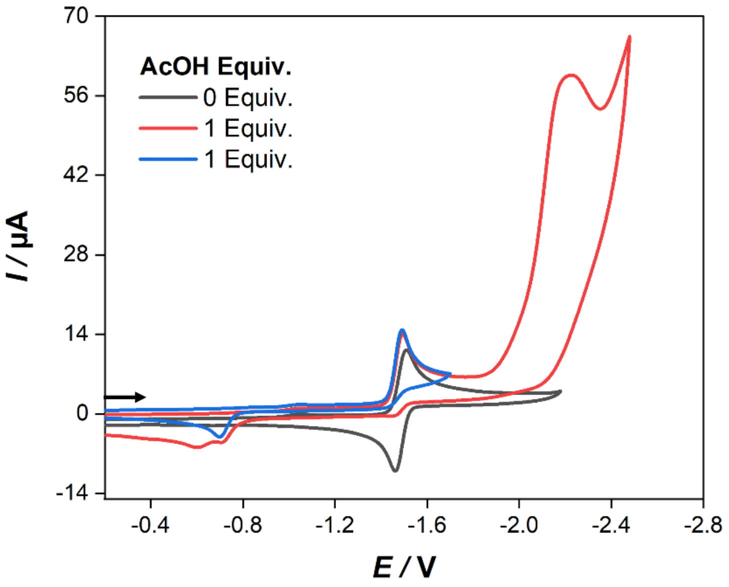

Figure S6. Cyclic voltammetry (0.2 V/s) of 1.0 mM of complex **3** in CH_2Cl_2– [*n*-Bu_4N][BF_4] (0.1 M) in the presence of one equiv. AcOH. Potential *E* is given in volts V and referenced to Fc^+/Fc couple. The arrow indicates the scan direction.

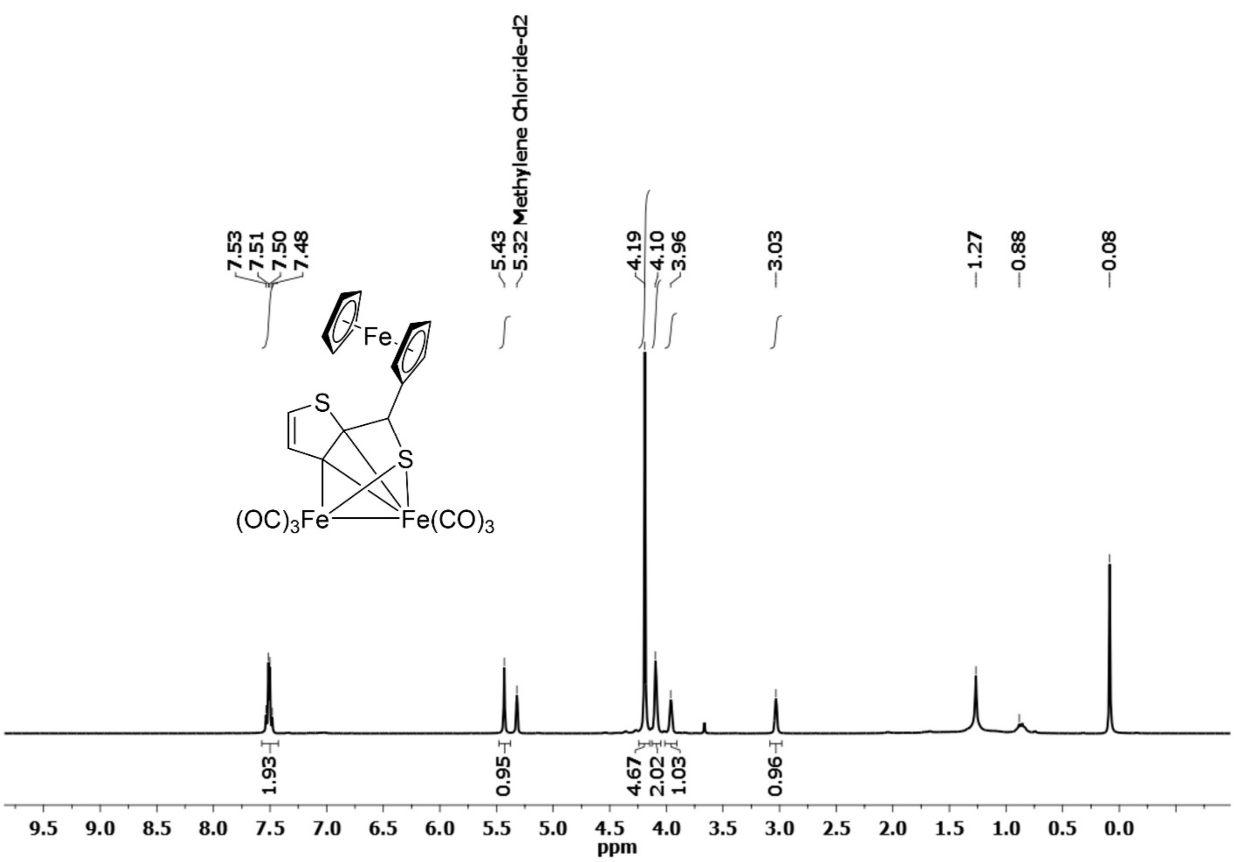

Figure S7. ^{1}H NMR spectrum of compound **2** in dichloromethane–d^{2} (400 MHz).

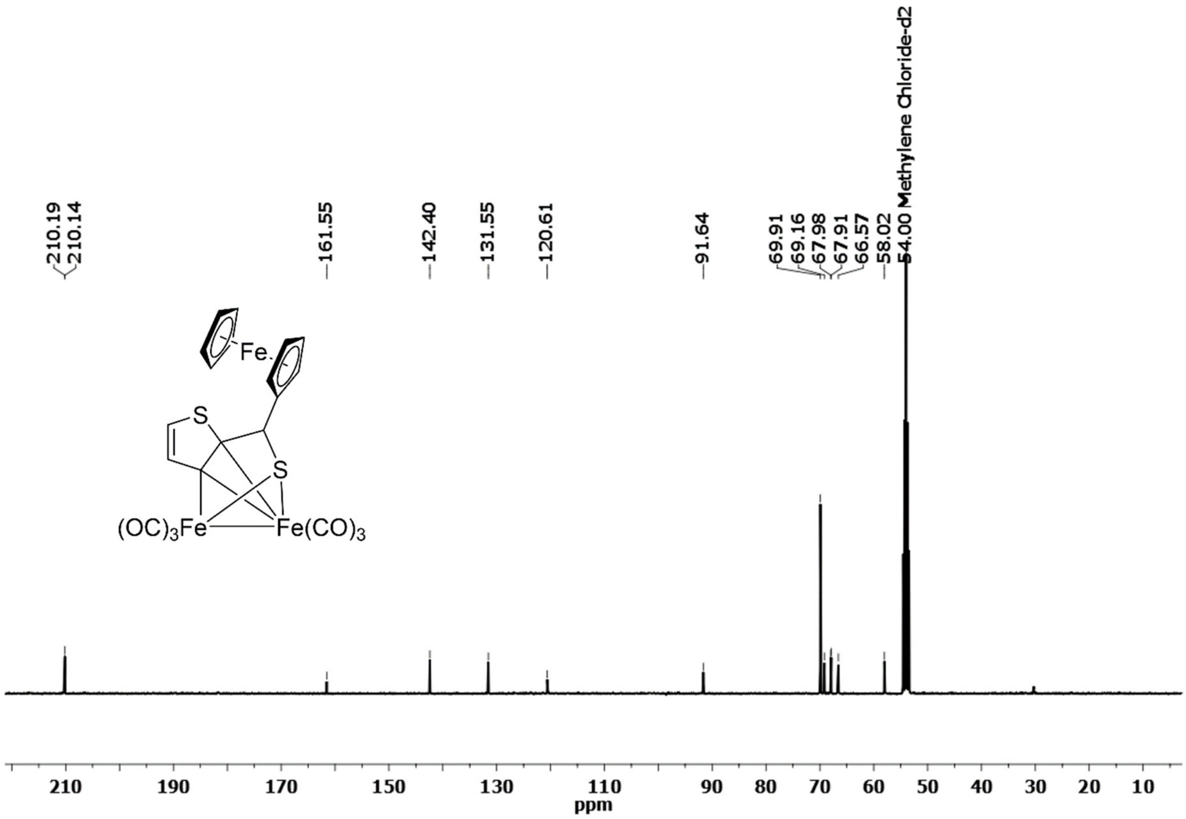

Figure S8. $^{13}C\{^{1}H\}$ NMR spectrum of compound **2** in dichloromethane–d^{2} (400 MHz).

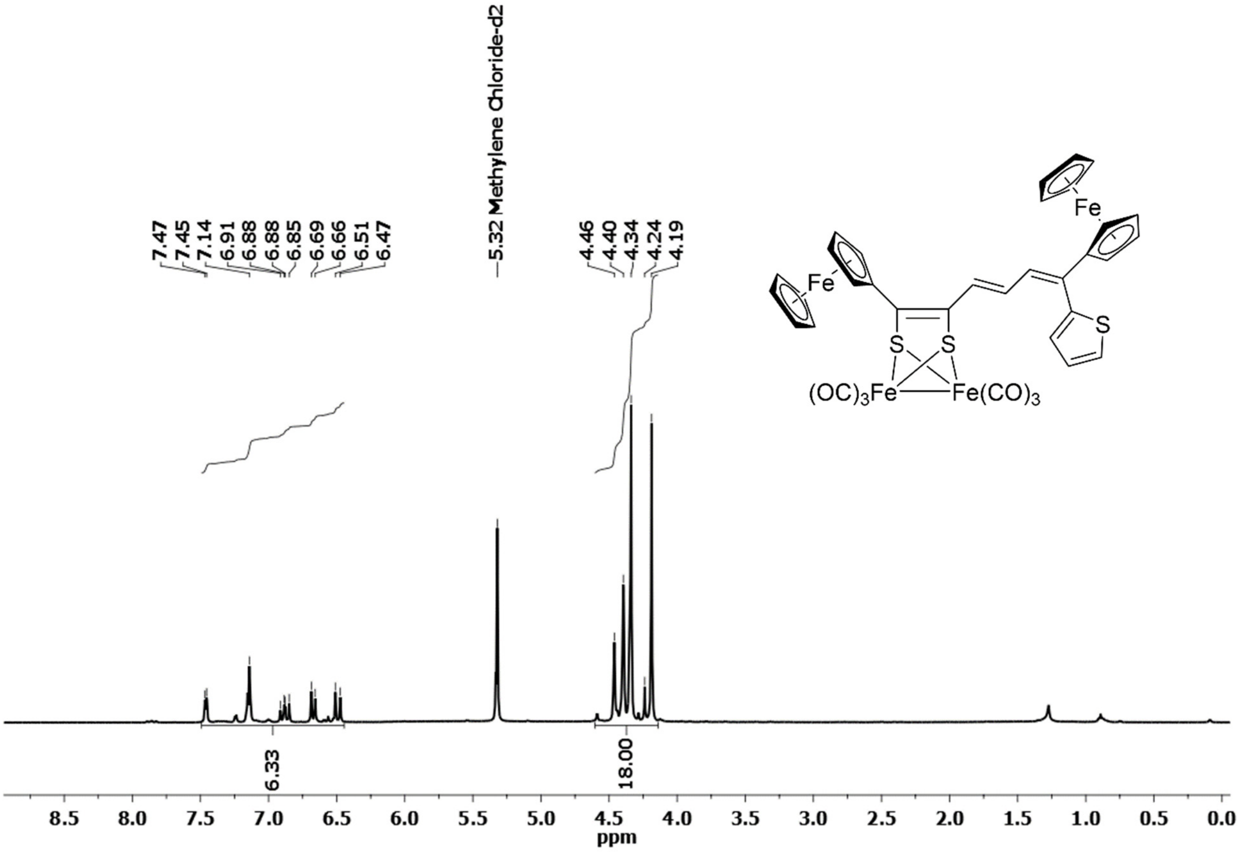

Figure S9. ^{1}H NMR spectrum of compound **3** in dichloromethane–d² (400 MHz).

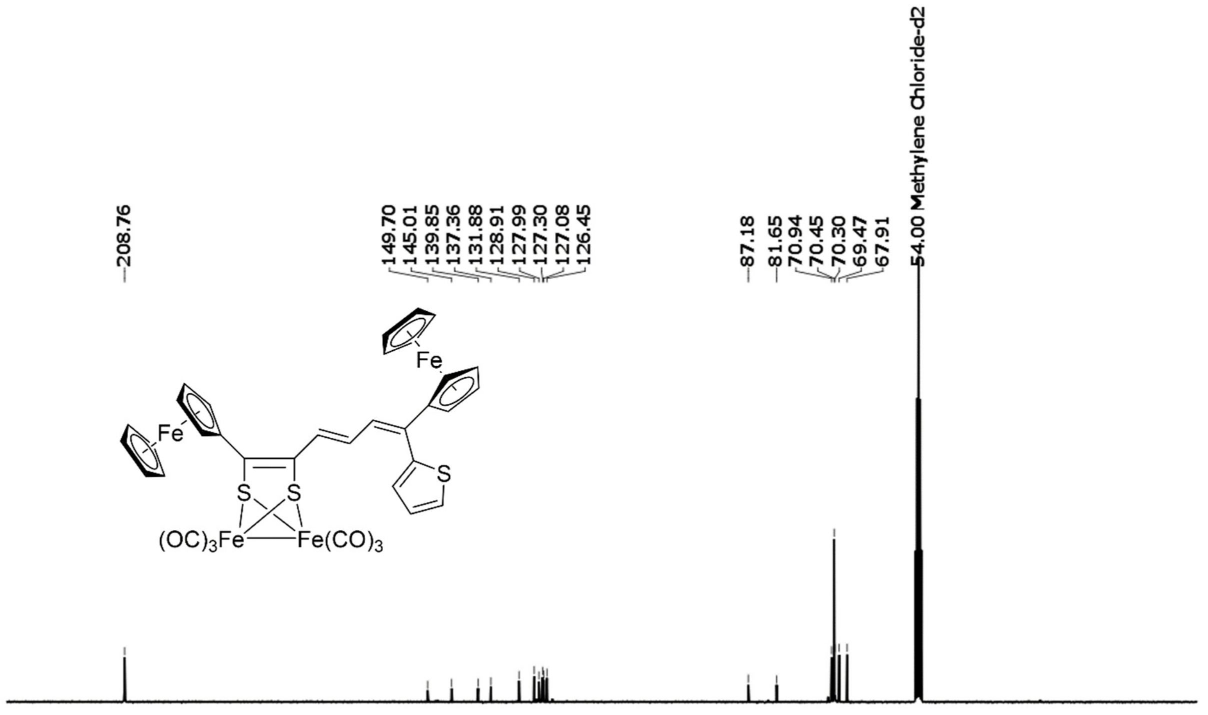

Figure S10. $^{13}C\{^{1}H\}$ NMR spectrum of compound **3** in dichloromethane–d² (400 MHz).

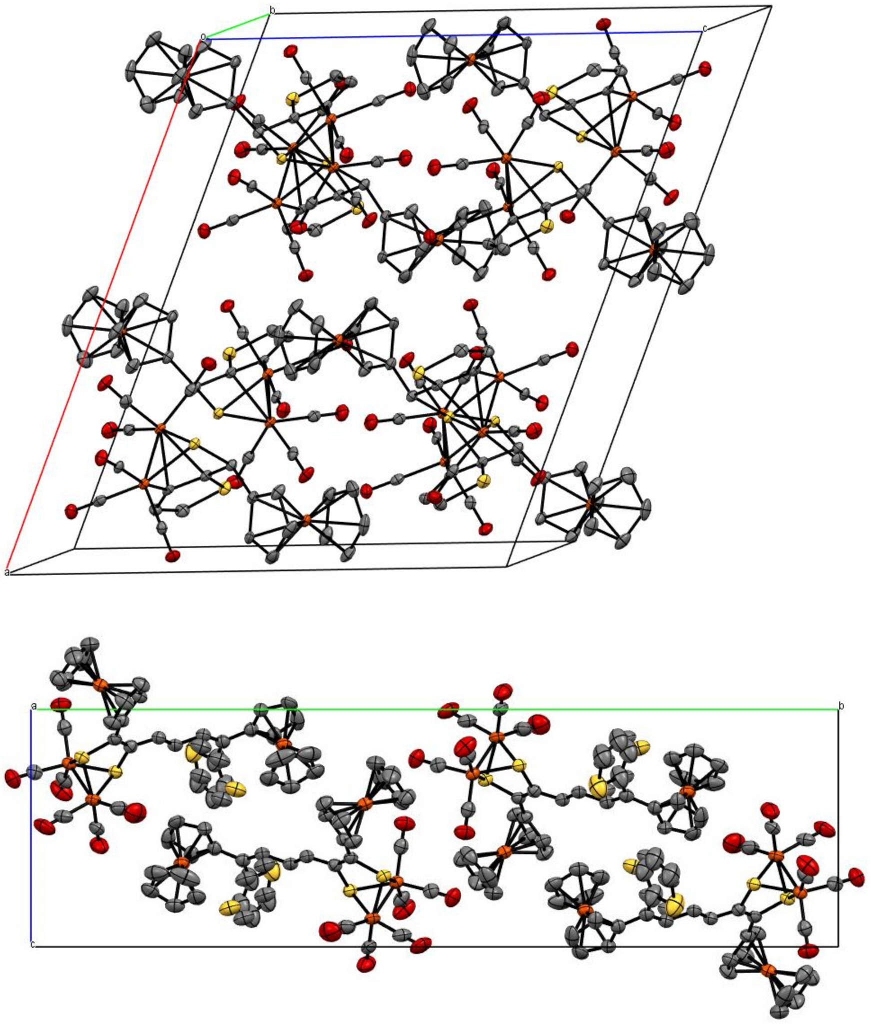

Figure S11. Packing diagrams of complexes **2** (**top**) and **3** (**bottom**).

References

1. Frisch, M.J.; Trucks, G.W.; Schlegel, H.B.; Scuseria, G.E.; Robb, M.A.; Cheeseman, J.R.; Scalmani, G.; Barone, V.; Petersson, G.A.; Nakatsuji, H.; Li, X.; et al. *Gaussian 16 Rev. C.01*; Gaussian, Inc.: Wallingford, CT, USA, 2016.
2. Beck, A.D. Density-functional thermochemistry. III. The role of exact exchange. *J. Chem. Phys* **1993**, *98*, 5648–5652.
3. Lee, C.; Yang, W.; Parr, R.G. Development of the Colle-Salvetti correlation-energy formula into a functional of the electron density. *Phys. Rev. B* **1988**, *37*, 785–789.
4. Dunning, T.H., Jr. Gaussian basis functions for use in molecular calculations. III. Contraction of (10s6p) atomic basis sets for the first-row atoms. *J. Chem. Phys.* **1971**, *55*, 716–723.
5. Dolg, M.; Wedig, U.; Stoll, H.; Preuss, H. A binitio pseudopotential study of the first-row transition metal monoxides and iron monohydride. *J. Chem. Phys.* **1987**, *86*, 2123–2131.
6. *COLLECT Data Collection Software*; Nonius B.V.: Delft, The Netherlands, 1998.
7. Otwinowski, Z.; Minor, W. Processing of X-Ray Diffraction Data Collected in Oscillation Mode. *Methods Enzymol*. **1997**, *276*, 307−326.
8. Krause, L.; Herbst-Irmer, R.; Sheldrick, G.M.; Stalke, D. Comparison of silver and molybdenum microfocus X-ray sources for single-crystal structure determination. *J. Appl. Cryst.* **2015**, *48*, 3–10.
9. Sheldrick, G.M. A short history of SHELX. *Acta Cryst.* **2008**, *A64*, 112–122.
10. Sheldrick, G.M. Crystal Structure Refinement with SHELXL. *Acta Crystallogr. Sect. CStruct. Chem.* **2015**, *C71*, 3–8.
11. Kratzert, D.; Krossing, I. Recent improvements in DSR. *J. Appl. Cryst.* **2018**, *51*, 928–934.
12. Macrae, C.F.; Edgington, P.R.; McCabe, P.; Pidcock, E.; Shields, G.P.; Taylor, R.; Towler, M.; van de Streek, J. Mercury: Visualization and analysis of crystal structures. *J. Appl. Crystallogr.* **2006**, *39*, 453−457.
